# Thermoplastic Starch-Based Blends with Improved Thermal and Thermomechanical Properties

**DOI:** 10.3390/polym13234263

**Published:** 2021-12-06

**Authors:** Anayansi Estrada-Monje, Sergio Alonso-Romero, Roberto Zitzumbo-Guzmán, Iván Alziri Estrada-Moreno, Erasto Armando Zaragoza-Contreras

**Affiliations:** 1 Centro de Innovación Aplicada en Tecnologías Competitivas, A.C. Calle Omega No. 201, Industrial Delta, León C.P. 37545, Mexico; salonso@ciatec.mx (S.A.-R.); rzitzumbo@ciatec.mx (R.Z.-G.); 2 Centro de Investigación en Materiales Avanzados, S.C. Miguel de Cervantes No. 120, Complejo Industrial Chihuahua, Chihuahua C.P. 31136, Mexico; ivan.estrada@cimav.edu.mx

**Keywords:** biobased blend, cassava starch, corn starch, polycaprolactone, thermoplastic starch

## Abstract

This research focused on the development of biomaterials based on cassava starch and corn starch and on the effect of the incorporation of polycaprolactone (PCL) on the thermal and thermomechanical properties of the blends. The results indicated partial compatibility in the blends, especially with cassava starch at a content of 20 wt% as reflected by the maintenance of tensile strength and elongation. In addition, the changes in the crystal quality of PCL and the displacement of the absorption bands of the carbonyl groups of PCL in the infrared (989–1000 cm^−1^), attributed to the formation of hydrogen bonds between these groups and the hydroxyl groups of starches, were also associated with compatibility. It was observed that the crystallinity of PLC in the presence of cassava and corn starch was 38% and 62%, respectively; a crystallinity greater than that of PCL was related to an improved nucleation at the interface. Based on these properties, the blends are expected to be functional for the manufacture of short-term use products by conventional thermoplastic processing methods.

## 1. Introduction

Plastics derived from petroleum have contributed to increasing the comfort of people’s everyday lives due to the notable versatility of properties that allows for the manufacturing of an infinite number of products. However, poor waste management and the lack of a culture of efficient recycling generate pollution that affects both terrestrial and marine ecosystems due to the accumulation of non-biodegradable plastic waste. For this reason, at the 4th United Nations Environment Assembly, a global agreement to reduce the consumption of non-biodegradable single-use plastics, was established [1]. With the growing need to take care of the environment and to find alternative materials that are more ecological for manufacturing short-term use products, research and development in biodegradable and bioplastic polymers has emerged [2].

Once in the environment, biomaterials biodegrade under the action of the weather and microorganisms [3]. The biodegradation products are carbon dioxide, methane, water, biomass, and several other harmless substances that are incorporated into the soil [4]. For example, the development of spoons made with biodegradable materials from seed flour, xanthan gum, and palm oil was performed as an alternative to the use of plastic cutlery [5].

Starches are essential raw materials for the development of biobased blends and composites, as they are natural and abundant biopolymers that come from grains such as corn, cassava, potato, rice, and others [6]. Native starch consists of two chemically different polysaccharides: amylose and amylopectin [7]. The former is a linear polymer composed of glucose units linked by α (1–4) bonds; despite some α (1–6) bonds that may be present, it is not water-soluble. The latter is a branched polymer of glucose units that are 95% linked by α (1–4) bonds, which is partially soluble in hot water [8]. According to Bertolini et al. [9], starches differ from each other by their amylose and amylopectin content, depending on the source of origin. García et al. [10] reported that the higher the amylopectin content, the higher the crystallinity in starches. For example, cassava starch is 17% amylose and 83% amylopectin, while maize starch is 28% amylose and 72% amylopectin [8]. Native starch has no plastic properties; however, when subjected to stress, under the action of temperature and in the presence of a plasticizer, a thermoplastic starch (TPS) can be obtained [11]. A TPS is meltable and processable by conventional processing methods. The plasticizer breaks the strong intramolecular interactions forming hydrogen bonds with the starch, resulting in plasticization [12]. Water, glycerol, sorbitol, sugar, ionic liquids, and compounds that have functional groups such as urea, formaldehyde, anhydride, or acetamide are applicable as plasticizers [13,14,15,16,17]. Although TPSs are biodegradable, they have poor mechanical properties and high susceptibility to water that restrict their use in many applications. One way to overcome these drawbacks is by blending with another biodegradable polymer that improves the mechanical properties of the final blend. Cellulose nanofibers and poly(vinyl alcohol) crosslinked with borax were used to improve the mechanical properties of starch for food packaging design [18]. Lactic polyacid has also been used in combination with starch, using hemp oil modified with maleic anhydride as plasticizer and compatibilizer. The added plasticizer showed improvements in the blend ductility and impact resistance [19]. Chitosan has also been used as a dispersed phase in TPS, incorporating it by thermomechanical methods and without the addition of compatibilizer. The addition of chitosan was found to considerably increase the Young’s modulus and tensile strength [20]. The addition of inorganic compounds for the mechanical reinforcement of TPS has also been reported. The addition of dolomite in a range of 1–5 wt% showed a better performance of tensile strength and Young’s modulus, especially for the higher inorganic contents. Better dispersion of the particles was also found to improve mechanical performance [21].

Polycaprolactone (PCL), a semi-crystalline and biodegradable thermoplastic characterized by its high flexibility, low melting point, and high compatibility with other polymers, has been explored to modify TPS [22,23]. For example, for a TPS plasticized with glycerol, it is possible to increase the blend’s hydrophobicity by incorporating PCL [24]. These types of blends have been studied for the improvement of their mechanical and thermal properties [13]. Cassava starch and PCL films, in the presence of an antioxidant, showed that the thermal properties of TPS were affected, which was interpreted as partial compatibility between polymers; however, tensile stress was reduced by 6% with the incorporation of PCL [25]. In another work, with the cassava starch–PCL blend, it was observed that the incorporation of PCL generated immiscibility, forming systems with starch as a dispersed phase, observed by electron microscopy. Furthermore, the addition of the PCL caused an increase in the elastic modulus and tensile stress at a relative humidity of 54% [26]. Considering the difficulties in integrating thermoplastic starch–PCL blends due to the polarity differences, the grafting of PCL on starch has been used. The grafting of the PCL was manifested as an increase in the flexibility of the TPS, without detriment to the biodegradability [27].

It is worth saying that although there are some studies dedicated to blends of PCL with TPS, there is still a lack of information on the properties of such blends and the analysis of their molecular interactions, so it is possible to improve the interfacial compatibility between the polymeric phases to improve the thermal and thermomechanical properties. Consequently, there is ample opportunity to contribute new knowledge on this topic. In this work, we investigated whether the type of starch (i.e., corn or cassava) influenced the generation of the best interaction towards a blend with improved properties without the use of coupling agents. We found evidence of the formation of an amylose–glycerol complex in blends with cornstarch, suggesting strong interactions between amylose and glycerol. The literature suggests that two opposite processes coexisted simultaneously in the blends: retrogradation, which stiffens the material, and plasticization, which softens it, with the latter mechanism predominating at short times and reversing at longer times. Moreover, there was evidence that the incorporation of PCL delays the retrodegradation, expanding the applications of the blend. It is important to highlight that conventional plastics transformation processes allow for exploring the possible applications of biomaterials to promote the use of ecological plastics, for example, in disposable plates and single-use cutlery.

## 2. Materials and Methods

### 2.1. Materials

Cassava starch (CaS) and corn starch (MaS) (Best Ingredients Mexico, Santa Catarina, Mexico), polycaprolactone (CAPA 6506, TRiiSO, Cardiff, CA, USA), and glycerol (CTR Scientific, Monterrey, Mexico) were used in the experimentation. All reagents were used as received.

### 2.2. Blend Processing

CaS and MaS plasticized with glycerol, hereinafter referred to as CaPS and MaPS, respectively, were prepared by manual mixing as follows: 70 wt% native starch and 30 wt% glycerol. Each system was fed into a twin-screw extruder (Micro27, Leistritz, Nuremberg, Germany) with an L/D of 32:1. The screw speed was 80 rpm, and the temperature profile was 110, 110, 120, 120, 130, 120, and 110 °C. The filaments obtained were air-cooled and pelletized.

Subsequently, the pellets of CaPS and MaPS were mixed with 20 wt% PCL. The blends were processed in the same twin-screw extruder at 80 rpm and a temperature profile of 110, 110, 120, 120, 130, 120, and 110 °C. At the end of the process, the blends were pelletized and referred to as CaPS–PCL and MaPS–PCL.

Finally, the blends were injected to obtain Type 1 specimens according to the ASTM D-638 procedure to evaluate the tensile strength and the percentage of elongation in universal traction equipment (model 5565 standard, Instron, Norwood, MA, USA).

### 2.3. Characterization

Functional groups were characterized using a Fourier-transform infrared spectrophotometer (Series 2 Magna IR 750, Thermo Fisher Nicolet, Waltham, MA, USA) in a wavenumber range of 4000–400 cm^−1^ with 16 scans and a resolution of 4 cm^−1^. 

The glass transition temperature (Tg) was determined using a differential scanning calorimeter (DSC, Pyris 1, Perkin–Elmer, Akron, OH, USA) under a nitrogen atmosphere, from room temperature to 200 °C at a heating rate of 10 °C min^−1^. The crystallinity index (CI) and the melting temperature of the PCL and blends were determined according to Equation (1) [28]:

(1) $$\mathrm{CI}\left(\%\right)=\left(\frac{\Delta \mathrm{Hexp}}{\Delta \mathrm{Hof}}\right)\times 100$$

where ΔHexp is the experimentally determined enthalpy of fusion (J g^−1^), ΔHo is the theoretical enthalpy of fusion of fully crystalline PCL (132 J g^−1^) [29], and f is given by the weight percentage of PCL in the mix.

Thermograms were obtained on a thermogravimetric balance (Q500, TA Instruments, DE, USA) in the temperature range 40–700 °C, with a heating rate of 10 °C min^−1^, a nitrogen purge flow of 20 mL min^−1^, and a sample of approximately 30 mg.

Dynamic mechanical analysis (RSA III, TA Instruments, New Castle, DE, USA) was achieved in the three-point mode at 1 Hz. Temperature sweeps were performed in the range −95–120 °C at a heating rate of 5 °C min^−1^. A strain of 0.1%, within the linear viscoelastic range, was chosen. The sample dimensions were 40 mm × 12 mm × 2 mm.

The mechanical properties were evaluated in universal tension equipment (model 5565 standard, Instron, MA, USA) according to the ASTM D-638 procedure. The tests were carried out at a rate of 50 mm min^−1^ with a 5 KN load cell at room temperature. Tensile strength (σ_max_) and elongation at break (ε) were evaluated.

### 2.4. Statistical Analysis

The Statgraphics^®^ 19 (Centurion XVI, The Plains, VA, USA) software was used for the statistical treatment of the data. Through its application, it was possible to carry out a descriptive analysis of one or more variables, using graphs that explained their distribution, calculating their characteristic measures as well as the calculation of confidence intervals.

## 3. Result

### 3.1. Analysis of Functional Groups

**Figure 1:**
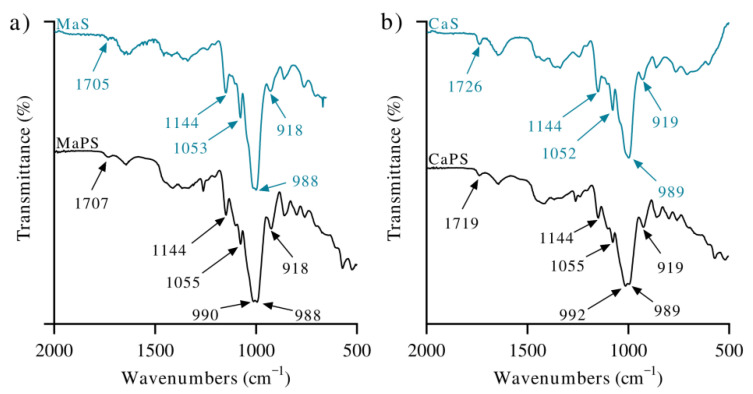
Infrared spectra of (**a**) native corn starch (MaS) and plasticized corn starch with 30 wt% glycerin (MaPS) and (**b**) native cassava starch (CaS) and plasticized cassava starch with 30 wt% glycerin (CaPS).

Figure 1a,b illustrate the FTIR spectra of MaS and MaPS and CaS and CaPS, respectively. Absorption bands in the range of 992–1200 cm^−1^ in starches were associated with interactions between starch molecules and the plasticizers [30]. The spectra of CaPS and MaPS showed the presence of a new peak at 992 and 990 cm^−1^, respectively; while the absorption of C–C–O of the anhydroglucosidic ring showed no change (988 cm^−1^), which appeared in the unplasticized starches MaS and CaS [31]. Interestingly, there was no displacement of the band at 988 cm^−1^ of C–C–O, but there was the formation of a new absorption, which Zullo et al. identified at approximately 1015 cm^−1^ [31], which suggests a new interaction as well as new modes of vibration after starch thermoplasticization. 

Another observation is that in CaS, there was a peak at 1726 cm^−1^ that can be associated with carbonyl groups. This signal shifted to 1719 cm^−1^ for the plasticized starch. It is worth mentioning that this peak appeared at lower values in MaS. A carbonyl signal was not expected in either of the two native starches, since no carbonyl group was generated during glycerol plasticization. However, the presence of this small peak was attributed to a starch decomposition process, which did not affect its physicomechanical properties [32].

Likewise, there was also an increase in the intensity of the absorption at 3362 cm^−1^, corresponding to the stretching of OH in plasticized starch. Importantly, this band was outstanding when there were intermolecular interactions as in the case of plasticized starch [33]. The weakening of the covalent bond force in which the donor H participated [34] was an attributed cause. 

**Figure 2:**
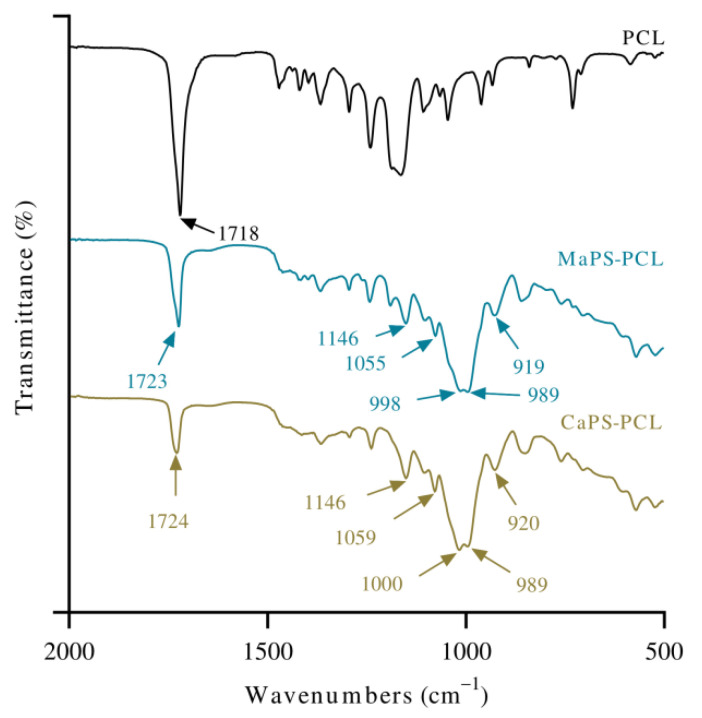
Infrared spectra of the blends CaPS–PCL and MaPS–PCL. PCL was included as a reference.

In the spectrum of MaPS–PCL, peaks corresponding to the PCL (Table 1), such as the absorption for C=O, suggest an interaction between both materials, whereas the absorption for OH at 3316 cm^−1^, with high intensity, indicates hydrogen bonding and intermolecular interactions. The displacement of the band at 989 cm^−1^ for MaPS and 992 cm^−1^ for CaPS (Figure 2) to 998 cm^−1^ (MaPS–PCL) and 1000 cm^−1^ (CaPS–PCL), respectively, manifests the interaction between PCL and starch. The functional groups that formed hydrogen bonds may be responsible in both cases for such displacements.

Mina et al. studied binary blends of plasticized cassava starch and PCL [33]. However, they used higher contents of PCL, finding poor miscibility between PCL and plasticized starch, evidenced by the lack of displacement in the absorption at 1723 cm^−1^ of PCL. Oppositely, at 20 wt% PCL, as in the present research, a displacement from 1718 cm^−1^ to 1724 cm^−1^ was found. This displacement raises the possibility of miscibility between the materials. Casteros reported the shift of infrared absorption bands with miscibility between polymers [34]. It is worth noting that some miscibility can arise in blends of starch and PCL because starch has proton donor groups (OH group) and PCL proton acceptor groups (carbonyl group) [26]. 

The shift in the carbonyl absorption was slightly more evident in CaPS–PCL, from 1718 to 1724 cm^−1^ (Figure 2), quite possibly because CaS contains more amylopectin than MaS. The functional groups that form hydrogen bonds may be more available in CaS as reported in the literature [8]. It has also been reported that in esters, the absorption of the carbonyl group presents a shift in the infrared absorption frequency when the group is associated [27].

### 3.2. Glass Transition and Crystallinity

**Figure 3:**
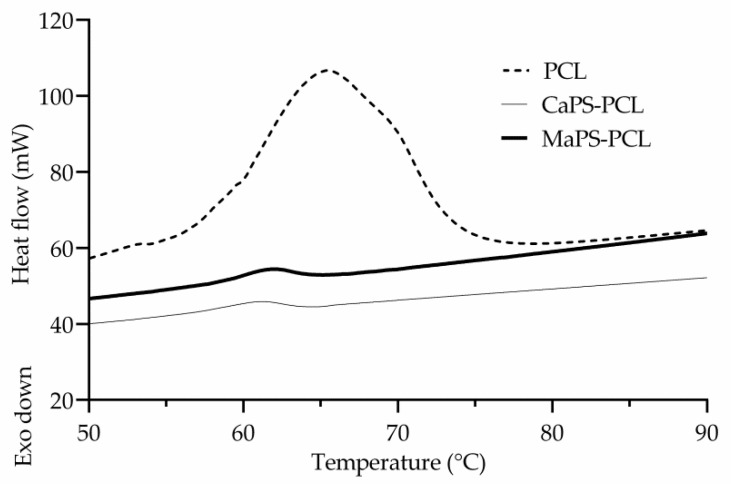
DSC thermograms of PCL, CaPS–PCL, and MaPS–PCL.

DSC was used to identify the transition temperatures and crystallinity index. Figure 3 shows the thermograms of PCL, MaPS–PCL, and CaPS–PCL. As observed, the PCL presented an intense transition at 65.4 °C, while the blends prepared with cassava and corn starch exhibited the endotherm at 61 °C and 61.5 °C, respectively. Since the content of the crystalline component (PCL) was low (20 wt%), the transitions were much less intense than in PCL. The continuous domain in the blends was plasticized starch, which probably caused the PCL crystals to be less packed and more heterogeneous, requiring less energy to melt [35]. Such a decrease in T_m_ seems related to compatibility between PCL and plasticized starches, as corroborated by the shift in the infrared absorptions in the blends as discussed before. It is worth mentioning that starch is thought to interfere in the crystallization process of the PCL, causing changes in the packaging and quality of the PCL crystals in the blends. Table 2 reports thermal properties and crystallinity of the blends and PCL. As seen, the CIs calculated from Equation (1) for MaPS–PCL and CaPS–PCL were 62% and 38%, respectively, indicating the crystallization of PCL was less affected by the presence of MaPS than CaPS, correlating with the infrared analysis in which a greater displacement in the absorption bands was found in blends with CaS than with MaS, due to the fact that CaS contained more amylopectin (~83%) than MaS (~72%). According to the literature, amylopectin has a branched structure and a much larger size than amylose, so the branched structure would probably interfere with the structural order of the PCL, manifesting itself in a low percentage of crystallinity.

### 3.3. Thermal Stability

**Figure 4:**
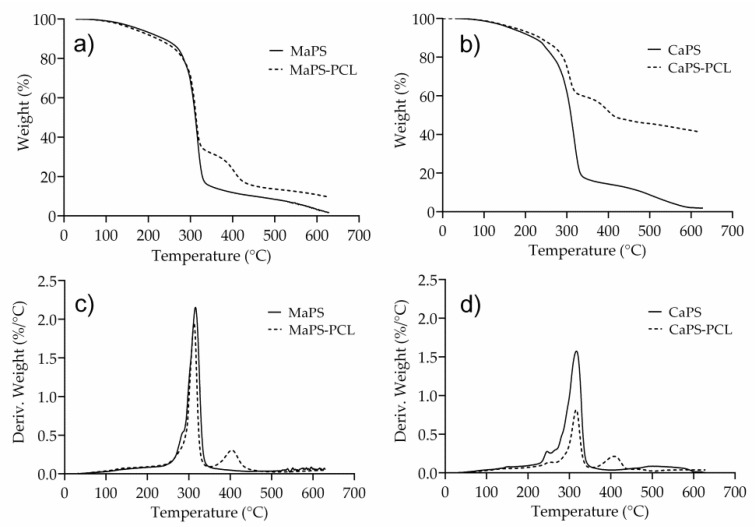
TGA traces of (**a**) MaPS and MaPS–PCL and (**b**) CaPS and CaPS–PCL. DTG traces of (**c**) MaPS and MaPS–PCL and (**d**) CaPS and CaPS–PCL.

Figure 4a,b show the TGA traces of the plasticized starches of cassava and corn and the blends with PCL. The curves exhibit an initial weight loss between 10 and 150 °C, corresponding to the evaporation of water and other volatiles present in the samples, in agreement with the literature [36]. Figure 4c,d show the DTA traces of the plasticized cassava and corn starches and the blends with PCL. The transition at approximately 250 °C in CaPS and CaPS–PCL (Figure 4d) corresponds to the dissociation of the amylose chains at high temperatures, which led to the structural breakdown [37]. This transition was not observed in MaPS and MaPS–PCL. It is known that amylose can form complexes with some alcohols, such as 1-butanol [37], which increases the thermal stability of amylose due to the formation of strong molecular interactions that cause more energy to be required to break the bonds. It is believed that in the case of MaS, which contained more amylose than CaS, an amylose–glycerol complex could have been generated, which increased the decomposition temperature of amylose, being reflected in a single transition in the thermogravimetric analysis, which corresponded to the decomposition of the amylose–glycerol complex and amylopectin.

The second decomposition event at 316 °C roughly matched the decomposition of amylopectin in CaPS and CaPS–PCL or the amylose–glycerol and amylopectin complex (Figure 4d) in MaPS and MaPS–PCL (Figure 4c), representing, in all cases, a significant weight loss. For CaPS–PCL, the decomposition of the PCL occurred at 408 °C, while for MaPS–PCL, the starch degraded at 312 °C and PCL at 404 °C. Consequently, the incorporation of PCL in the thermoplastic starches did not affect the decomposition temperature profile of the starches; thus, CaPS and MaPS showed similar behavior with significant weight losses at the same temperatures.

### 3.4. Dynamic Mechanical Analysis

**Figure 5:**
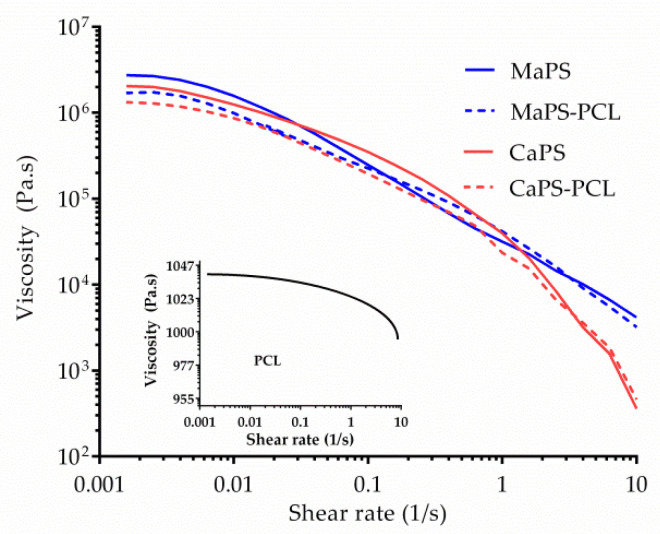
Viscosity of the starch composites as a function of shear rate at 130 °C. Inset: viscosity of polycaprolactone under the same conditions.

Figure 5 shows the effect of the incorporation of PCL on the viscosity of the blends with corn and cassava starch. As seen, PCL had a plasticizing action, since at a zero shear rate, MaPS and CaPS had a higher viscosity than the MaPS–PCL and CaPS–PCL blends.

**Figure 6:**
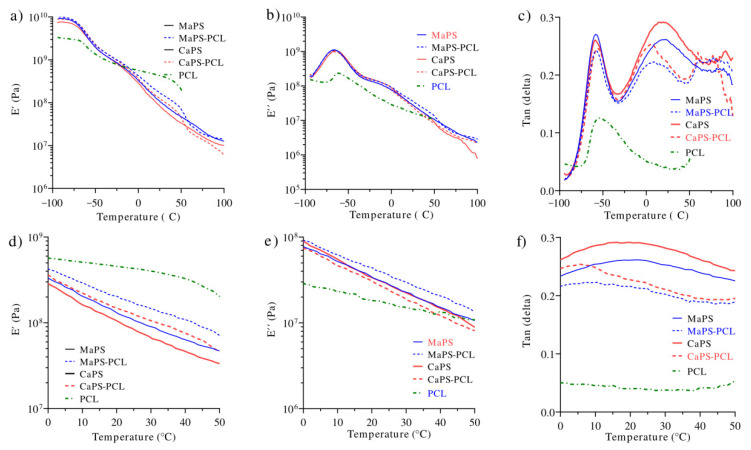
Loss factor (Tan delta) of the blends as a function of temperature.

To evaluate the influence of PCL on the thermal properties of thermoplastic starches, DSC and DMA were used. Because thermoplastic starches consist of two phases, one rich in starch and another rich in glycerol, transitions for both domains could be expected. Thus, the Tg corresponding to the glycerol-rich phase appeared at −57 °C for all materials including blends with PCL (Figure 6c). In this same temperature interval, the Tg of the PCL also appeared [38]; thus, there could be an overlap of both Tgs. On the other hand, the Tg corresponding to the phase rich in starch appeared at approximately 20 °C.

The materials exhibited pseudoplastic behavior since the viscosity decreased with increasing shear deformation. Said flow behavior was also called shear thinning, which is associated with an increase in the degree of orientation of the polymeric molecules and the deterioration in the interlacing of chains, both in thermoplastic starches and PCL. Furthermore, the thermoplastic starches and the blends with PCL presented much higher viscosities than pure PCL (Figure 5). Correa et al. observed similar behavior in viscosity measurements made at 110 and 130 °C [36]. The addition of PCL in the thermoplastic starches caused a slight decrease in the blend viscosity, which resulted in the blends being slightly less viscous than the thermoplastic starch alone. The graph indicates that the plasticized cassava starch blended with PCL (CaPS–PCL) presented a slightly lower viscosity than the maize material (MaPS–PCL), probably explained in terms of the amylopectin content in the cassava starch. As observed by DSC, CaPS–PCL had a lower structural order, so it would be expected that the interactions would break down at lower temperatures than in the blend with cornstarch.

Figure 6a shows the storage modulus (E’) of the blends in a wide range of temperatures in which similar performances were observed for all of the blends. However, in the temperature range of 20–40 °C, which is where the majority of practical applications occur (Figure 6d), the MaPS–PCL and CaPS–PCL blends were the ones with the highest rigidity, below the PCL, which decreased as the temperature increased. According to this, above 40 °C, the material would not be functional for applications that require a polymer with greater rigidity.

Figure 6b depicts the behavior of E”, which was consistent with E’, indicating a similar behavior in all of the blends. In the range of 20–40 °C (Figure 6e), the blends did not present a significant difference, concluding that the application temperature of the materials should be between 20 and 40 °C to achieve good mechanical performance. 

In Figure 6a,c, transition was present at approximately 3 °C for MaPS–PCL and at 6 °C for CaPS–PCL, which was only present in the blends of thermoplastic starch with PCL. It seemed to be caused by the formation of a more rigid component present in the blends, causing the appearance of transitions above 70 °C; however, further research is required to corroborate its structure and influence on blends.

### 3.5. Mechanical Properties

The mechanical properties of the CaPS and MaPS and CaPS–PCL and MaPS–PCL were evaluated by strain–stress tests. The tensile strength values reported as maximum tensile strength, σ_max_, and elongation at break are shown in Table 3.

The method currently used to discriminate between means was the Fisher’s least significant difference (LSD) procedure. With this method, there is a risk of 5.0% when saying that each pair of means is significantly different when the real difference is equal to 0 (Table 3).

A multiple comparison procedure was applied to determine which means were significantly different from others. Table 4 shows the estimated differences between each pair of means. The asterisk next to the four pairs indicates that these pairs showed statistically significant differences with a 95% confidence level.

Multiple range tests were performed in Statgraphics, reporting the following results: The Kruskal–Wallis test evaluated the hypothesis that the medians of the tensile strength of the materials within each of the four levels of the compound were equal. Since *p* < 0.05, there was a statistically significant difference among the medians with a 95% confidence level (Table 5).

The results indicate a significant difference in the blend tensile strength when corn or cassava starch was used and when including PCL in the blend. Probably, the susceptibility to water increased with the incorporation of PCL; however, this was not evaluated in the present work.

Other investigations have focused on plasticized starch, trying to modify some characteristics such as water absorption, resistance to tension, or modification of Tg. Hernandez-Medina et al. reported that by acetylation, it was possible to decrease Tg and water absorption; however, tensile strength also decreased compared to natural TPS [8]. Mahieu et al. obtained blends of TPS with PCL of different molecular weights [39]; they found a decrease in deformation that was attributed to the lack of compatibility between the two components when 30 wt% PCL was used. In the case of Correa et al., various contents of PCL were blended with TPS, finding that the mechanical properties improved compared to pure TPS. In addition, because the blends are fully biodegradable, they can be used as vehicles for the controlled release of nutrients into the soil while they biodegrade [36]. As previously stated, the deformation of the TPS was not affected by the presence of PCL, since partial compatibility was found between the materials, at least with a load of 20 wt% of PCL.

It is worth mentioning that the retrogradation of starch in blends of TPS and PCL has been studied less thoroughly. In the work of Mina et al., in which 40–60 wt% of PCL was used, it was found that the blends were predominantly immiscible, considering that the incorporation of PCL in the retrogradation process of the blends was negligible [33]. However, due to the structure of starch and PCL, the formation of hydrogen bonds and secondary interactions that could intervene in the structural changes within the TPS would be expected.

TPSs are a new class of green polymers that can be disposed of after use without contamination. However, there are still some little-studied aspects that should be of importance in future research to create new products to serve human society. The limitations of the use of TPSs are their high sensitivity to moisture and retrogradation processes. Therefore, more studies are required for starch (i.e., native and modified) and plasticizers that focus on reducing water absorption and material retrogradation and, thus, obtaining materials with improved mechanical properties for various applications [40]. In this work, the properties exhibited by the blends can be useful for the manufacture of short-lived products such as disposable utensils and plates.

## 4. Conclusions

In this work, bio-based blends derived from corn and cassava starch with PCL were developed. FTIR and calorimetry showed partial compatibility between thermoplastic starch and PCL, manifested by the shift of absorption signals and the melting temperature of PCL. Consequently, a better mechanical performance of the blends was attained. Thermogravimetric analysis showed evidence of the formation of an amylose–glycerol complex in blends with cornstarch that increased the decomposition temperature of amylase, moving to overlap with the decomposition temperature of amylopectin, presenting a single transition. Plasticized corn starch and cassava starch blended with PCL exhibited appropriate extrusion and injection processability and low susceptibility to water. Based on the present research, the next step is to quantify the decrease in the susceptibility to water of the blends, determine the biodegradability time under controlled conditions, in natural and accelerated weathering, to determine more precisely the performance of the blends in the design of single-use utensils such as cutlery and disposable plates.

## 5. Patents

Patent application MX/a/2020/011248. Biopolímeros y método para su preparación (Biopolymers and method for their preparation). By Anayansi Estrada-Monje, Sergio Alonso-Romero, and María Maldonado-Santoyo. October 2020.

## Figures and Tables

**Table 1:** Band assignation in the FTIR spectra.

Bonding	Wavenumbers (cm^−1^)					
	Experimental Values		Mina et al. [33]	PCL	MaPS	CaPS
	MaPS–PCL	CaPS–PCL				
O–H stretching	3270	3270	3331		3288	3270
Asymmetric/symmetric CH_2_/CH_3_	2929/2872	2930/2870	2945/2866	2942/2866	2913/2887	2920/2887
C=O Stretching (PCL)	1723	1724	1724	1718		
Asymmetric stretching C–O–C (PCL)	1242	1242	1242		1242	1242
Stretching of Glycosidic C–O–C	1055/1146	1059/1146	1043/1029		1055/1144	1055/1144
Water absorption	1622	1621			1618	1625
Glycosidic bond, C–O stretching	989	989			988	989
After plasticization	998	1000			990	992

**Table 2:** Crystallization temperature (Tc), melting enthalpy (∆Hc), and percentage of crystallization (C_I_) of PCL, MaPS–PCL, and CaPS–PCL.

Sample	T_m_ (°C)	ΔH_m_ (J g^−1^)	T_c_ (°C)	ΔH_c_ (J/g)	C_I_ (%)
CaPS–PCL	61.0	10.0	-	-	38.0
MaPS–PCL	61.5	16.5	-	-	62.0
PCL	16.6	39.7	65.4	233	28.5

**Table 3:** Tensile strength and percentage of elongation of the developed materials.

Material	σ_max_ (MPa)	Elongation (%)	Cases	Media	Standard Error	Homogeneous Groups
MaPS	0.210 ± 0.019	63	6	0.1642	0.010219	X
CaPS	0.164 ± 0.005	90	6	0.2101	0.010219	X
MaPS–PCL	0.168 ± 0.003	60	6	0.1680	0.010219	X
CaPS–PCL	0.227 ± 0.040	70	6	0.2272	0.010219	X

**Table 4:** Contrast between materials.

Contrast	Significance	Difference	+/− Limits
MaPS–MaPS–PCL	*	0.0421	0.0301
MaPS–CaPS	*	0.0458	0.0301
MaPS–CaPS–PCL		−0.0171	0.0301
MaPS–CaPS		0.0037	0.0301
MaPS-PLC–CaPS–PCL	*	−0.0592	0.0301
CaPS–MaPS–PCL	*	−0.0629	0.0301

* Significant difference.

**Table 5:** Kruskal–Wallis test for the tensile strength of the materials.

Material	Sample Size	Average
MaPS	6	17.16
MaPS–PCL	6	8.50
CaPS	6	6.50
CaPS–PCL	6	17.83

Statistic = 12.2667, *p*-value = 0.0065.

## Data Availability

The data presented in this study are available on request from the corresponding author. The data are not publicly available due to privacy.
